# Adherence to Stillbirth Guidelines and Women's Psychological Well‐Being: The CLASS (CiaoLApo Stillbirth Support) Cross‐Sectional Study

**DOI:** 10.1111/birt.70087

**Published:** 2026-06-19

**Authors:** Claudia Ravaldi, Francesca Roper, Laura Mosconi, Alfredo Vannacci

**Affiliations:** ^1^ CiaoLapo Foundation for Perinatal Health Prato Italy; ^2^ PeaRL—Perinatal Research Laboratory, Department of Neurosciences, Psychology, Drug Research and Child Health University of Florence Florence Italy

**Keywords:** bereavement care, grief, guidelines, PTSD, respectful care, stillbirth

## Abstract

**Background:**

The psychological impact of stillbirth on parents is profound, increasing the need for respectful care. Despite the existence of international guidelines, there has been no clinical confirmation of their efficacy in improving parental mental health outcomes.

**Methods:**

This study is a web‐based cross‐sectional study and part of the OPALE (Observatory on PerinatAL hEalth) project. Participants were selected if they suffered a pregnancy loss after 20 weeks (including termination of pregnancy for medical reasons) in the last 10 years. The survey includes: the CiaoLapo Stillbirth Support (CLASS) checklist, the Perinatal Grief Scale (PGS), the National Stressful Events Survey PTSD Short Scale (NSESSS), and questions on satisfaction with care.

**Results:**

261 participants completed the survey. In a multivariate analysis, higher CLASS scores were correlated with lower PGS and NSESSS scores, suggesting a direct relationship between guideline adherence and better psychological outcomes. Specifically, satisfying over 40 of the 60 checklist items independently predicted greater care satisfaction (OR 2.0, CI 1.1–3.8), higher experiences of respectful care (OR 3.6, CI 1.9–7.0), lower grief (OR 0.08, CI 0.1–0.2), and reduced PTSD symptoms (OR 0.21, CI 0.1–0.5).

**Conclusions:**

This is the first study which identifies a correlation between adherence to stillbirth care guidelines and better psychological outcomes, indicating their importance in enhancing parents' mental health.

## Graphical abstract

CLASS checklist adherence was evaluated in a web‐based survey of 261 women who experienced stillbirth in Italy. Greater adherence to evidence‐based stillbirth care guidelines was associated with higher satisfaction and respectful care, as well as lower levels of perinatal grief and PTSD symptoms. These findings support the implementation of local guidelines and professional training to improve bereavement care and maternal psychological outcomes after stillbirth.

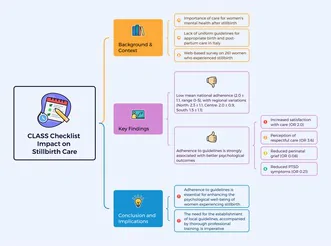

## 1 Introduction

Annually, around 2.6 million pregnancies worldwide result in stillbirth, and the majority of these are preventable [1]. Stillbirth often remains an overlooked global health concern, with many countries lacking comprehensive public health strategies to mitigate preventable deaths or to systematically gather and analyze quality data on such tragic events [2]. In Italy, the stillbirth rate is around 2.4 for every 1000 births with a 15.1% reduction from 2000 to 2019. However, a high rate disparity between Southern and Northern regions remains [3].

The repercussions of stillbirth extend beyond mortality rates and physical health impacts on women to profoundly affect mental health as well [4, 5]. Experiencing a stillbirth is a deeply traumatic event for couples [6], and without supportive and respectful care, it can result in significant psychological distress, increasing societal burdens [7]. Yet, the provision of equitable, high‐quality bereavement care remains an under‐addressed aspect of health services in many regions [4]. Furthermore, the task of delivering bereavement care can negatively impact healthcare professionals (HCPs), potentially leading to professional burnout when they are untrained, emotionally unprepared, or left to manage these challenging situations on their own [8].

Acknowledging the profound impact of stillbirths on both families and HCPs, there is a pressing need for guidelines to enhance the quality of post‐stillbirth care [8, 9]. Initial research on the clinical management of stillbirths, aimed at facilitating the natural grieving process, emerged in the 1970s [10]. Since then, several countries have developed national guidelines to better support birthing people and their partners experiencing stillbirth [11, 12, 13, 14]. These guidelines have been recently synthesized in the CiaoLapo Stillbirth Support (CLASS) checklist—a tool categorizing the guidance from various national protocols into six key areas: respect, information and communication, choices surrounding birth, hospital stay, creating memories, and subsequent care [9, 15].

The World Health Organization has highlighted the need for respectful care and the establishment of official national guidelines to support HCPs in delivering bereavement care [5, 16]. Despite these recommendations, many countries lack official national guidelines, leaving HCPs without formal direction. Recently, Italian recommendations for the clinical management of stillbirth were published thanks to a collaborative effort by the leading Italian obstetricians' associations [17]. While these mark a significant step towards more respectful bereavement care, no formal guidelines endorsed by the Italian National Health Service are currently available, leaving the implementation of these recommendations in clinical practice at the discretion of individual HCPs.

Since its founding in 2006, the Italian charity CiaoLapo has provided over 800 h of formal training to HCPs across 100 maternity units, representing 20% of all Italian maternity units [18]. A significant number of these units have implemented the bereavement care guidelines proposed by CiaoLapo and summarized in the CLASS checklist, leading to reported enhancements in stillbirth care. Parents receiving care after HCPs underwent CiaoLapo's training have expressed higher satisfaction compared to those assisted before such training was available [9, 19, 20]. Despite these advancements, challenges remain: many units lack specific local bereavement care protocols.

Considering the variability in the distribution of guidelines across Italy and the limited evidence reagrding their effectiveness, the CLASS study had three main objectives: (1) verify the impact of international guidelines about perinatal bereavement care on parents' perspectives about care and psychological well‐being; (2) assess the current state of bereavement care in Italy; and (3) determine if adherence to guidelines is correlated with improved parental outcomes.

## 2 Methods

This study is part of the permanent OPALE project (Observatory on PerinatAL hEalth) and follows a cross‐sectional study design. The survey is hosted on the Qualtrics platform provided by Florence University PeaRL laboratory and is distributed through the online channels of CiaoLapo Foundation, an organization working on the promotion of perinatal health in Italy. Data were collected from the beginning of 2021 to the end of 2022. The survey was voluntary and anonymous; no personal data were recorded, making it impossible to identify any single respondent. Informed consent was obtained from all participants. The data were acquired in adherence with GDPR regulations (General Data Protection Regulation, European Union 2016/679). Information about the questionnaire was provided on the first page of the survey, and consent was given by participants before proceeding with the survey. Participants were selected for the CLASS study if they had suffered a pregnancy loss after 20 gestational weeks (including termination of pregnancy due to pathology) in the last 10 years. The uneven adoption of different practices throughout Italy provides a unique opportunity to compare the experiences of parents who received care in alignment with the guidelines to those who received different forms of care while controlling for other concurrent variables.

The following sections of the OPALE survey were used for the CLASS study:

1. Socio‐demographic information and medical history.
2. The CLASS checklist, with 60 items common to the main international guidelines on bereavement care [11, 12, 13, 14], covering the following six areas:
  - Respect for the baby and parents, that is, naming the baby, bathing and dressing the baby, providing privacy, allowing partners to spend time together, etc.
  - Information and communication, that is, using parent‐friendly language, avoiding dehumanizing terms and medical jargon, giving written information, discussing issues at the most appropriate time in a quiet and private environment, etc.
  - Birth options, that is, offering parents choices, offering the option of returning home, offering obstetric analgesia, avoiding sedation, etc.
  - Hospital stay, that is, providing privacy, allowing time with the baby, not urging parents to leave the hospital, etc.
  - Creating memories, that is, allowing parents to see and hold their baby, providing mementos such as a lock of hair, footprints, ID bracelet, etc.
  - Aftercare, that is, informing mothers about physical and psychological consequences of perinatal loss, providing early psychological support, providing written information on support services, discussing implications for future pregnancies, etc.
  The CLASS checklist was used as a measure of adherence to international stillbirth guidelines. Parents were asked to rate every item from 0 (not respected at all) to 4 (fully respected) or to select the option “I don't know or not applicable”. Items were considered “satisfied” with a score equal or greater than 2. The CLASS checklist is available online in Italian and English (www.class.ciaolapo.it); the English version is provided here as Table S1.
3. The Perinatal Grief Scale (PGS), an instrument used to assess grief after perinatal loss [21, 22]. The scale consisted of 33 Likert‐type questions, with answers ranging from 1 (strongly agree) to 5 (strongly disagree). The PGS presents three subscales: ‘active grief’ (AG), ‘difficulty in coping’ (DC), and ‘despair’ (D). The PGS total score has a possible range of 33–165; each subscale has a possible range of 11–55.
4. The National Stressful Events Survey PTSD Short Scale (NSESSS), a 9‐item measure that assesses the severity of posttraumatic stress disorder in individuals aged 18 and above following an extremely stressful event or experience [23]. Each item asks the individual receiving care to rate the severity of his or her posttraumatic stress symptoms during the past 7 days. Each item is rated on a 5‐point scale (0 = Not at all; 1 = A little bit; 2 = Moderately; 3 = Quite a bit, and 4 = Extremely). The total score can range from 0 to 36 with higher scores indicating greater severity of posttraumatic stress symptoms.
5. The survey also included items assessing women's satisfaction with care and women's perception of respectful care. Items were rated from 1 (not satisfied at all) to 5 (fully satisfied) and then classified as yes/no when needed for dichotomous outcomes analysis [24, 25].

### 2.1 Statistical Analysis and Data Presentation

Survey responses were extracted from the OPALE dataset hosted in Qualtrics platform and imported into Stata/BE 17.0 (StataCorp) for statistical analysis. Categorical data were reported as frequencies and percentages and compared using the chi‐squared test, whereas continuous data were reported as mean values with standard deviations (SD) or as median [quartiles] and compared using *t*‐test or Kruskall Wallis and Mann Witney test. All results were considered statistically significant at *p* < 0.05.

Four different multivariate analyzes were performed to evaluate the association between the binary outcomes—(a) satisfaction of care, (b) perception that the care was respectful, (c) higher quartile of PGS total score (> 103), and (d) higher quartile of NSESSS score (> 22)—and the following variables: maternal age, medical diagnoses, previous psychopathology, previous losses, pregnancies after loss, gestational age at loss, months elapsed after loss, and number of CLASS items satisfied. Responders' locations were mapped by regional areas across Italy; scores, correlation graphs, and box plots were plotted using Tableau Desktop 2023.1 (Tableau Software LLC). A mind map of key findings (graphical abstract) was created with the software Xmind (v23.08 Xmind LTD).

## 3 Results

### 3.1 Sociodemographic Information and Medical History

The sample consisted of 261 women, with the majority (70.9%) being under 40 years old; 25.3% of respondents experienced loss less than 12 months prior to answering the questionnaire. The gestational age at loss was equally distributed between 20–25 weeks (33.3%), 26–36 weeks (35.2%), and 37–42 weeks (31.4%); 14.0% of them reported a termination of pregnancy for medical reasons. Over half of the women (54.0%) had subsequent pregnancies, and 10.7% were currently pregnant. In terms of medical history, coagulopathy (11.5%) and obesity (14.6%) were the most common disorders reported, while anxiety was the most prevalent psychological history (23.4%). Finally, most women (72.8%) experienced only one loss, while 17.2% experienced 2–3 losses, and 10% experienced more than three. Full details of the sample are reported in Table S2.

### 3.2 Adherence to the CLASS Checklist

The mean score of mothers' perception of adherence to guidelines was 2.0 (SD 1.1; range 0.2 to 4.0; median value 1.9 [1.0; 3.0]). A significant difference between regions was observed, with Northern Italy scoring 2.32 [1.26; 3.38], Central Italy 2.00 [1.08; 2.92], and Southern Italy 1.48 [0.40; 2.56] (Figure 1).

**FIGURE 1:**
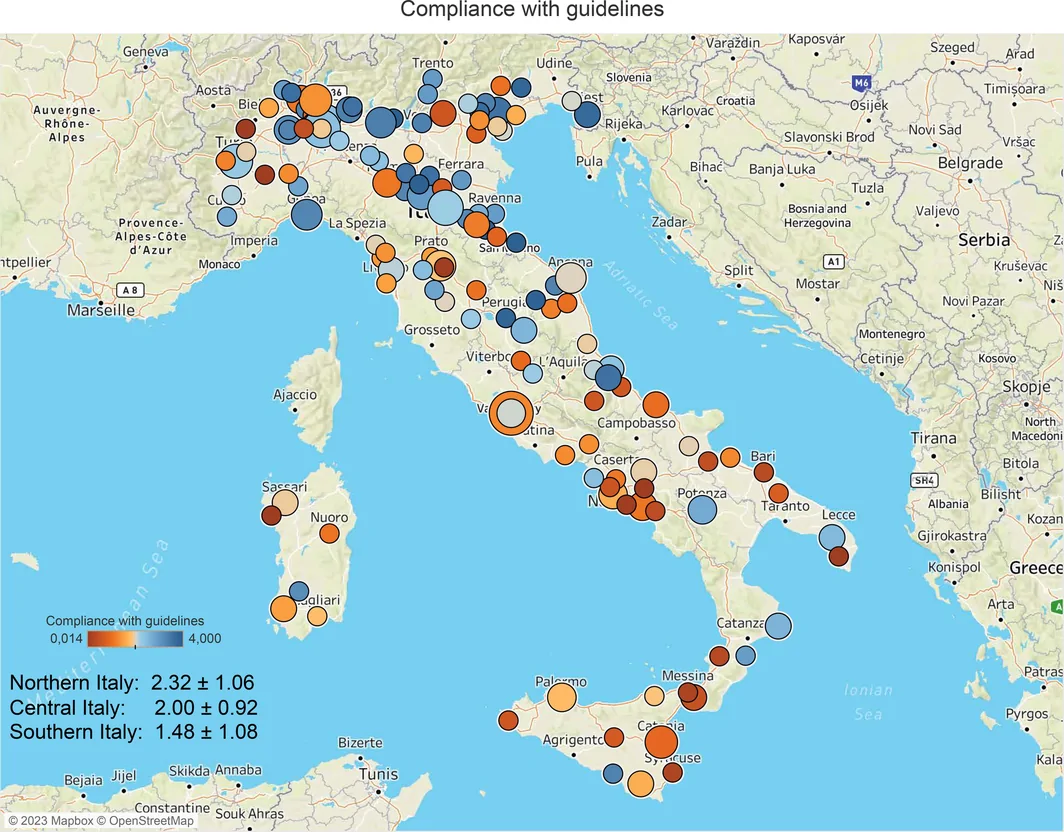
Adherence to international guidelines about perinatal bereavement care: A map of responders. [Colour figure can be viewed at wileyonlinelibrary.com]

Mean scores of CLASS subcategories were 1.96 on a 4‐point Likert scale, with the highest value (2.54, SD 1.2) in the “Mode of Birth” (Cb) section. Categories with ratings lower than the mean score were: (A) respect, specifically towards the baby (Aa); (B) communication, specifically about the baby's funeral (Be) and autopsy (Bf); (E) creating memories, particularly in relation to spending time with the baby (Ea), parenting (Eb), and having mementos (Ec); and (F) aftercare, with regards to maternal changes (Fb), support services (Fc), and follow‐up (Fd), with the lowest score reported in “Parenting” (Eb) section (0.61, SD 1.2) (Figure 2).

**FIGURE 2:**
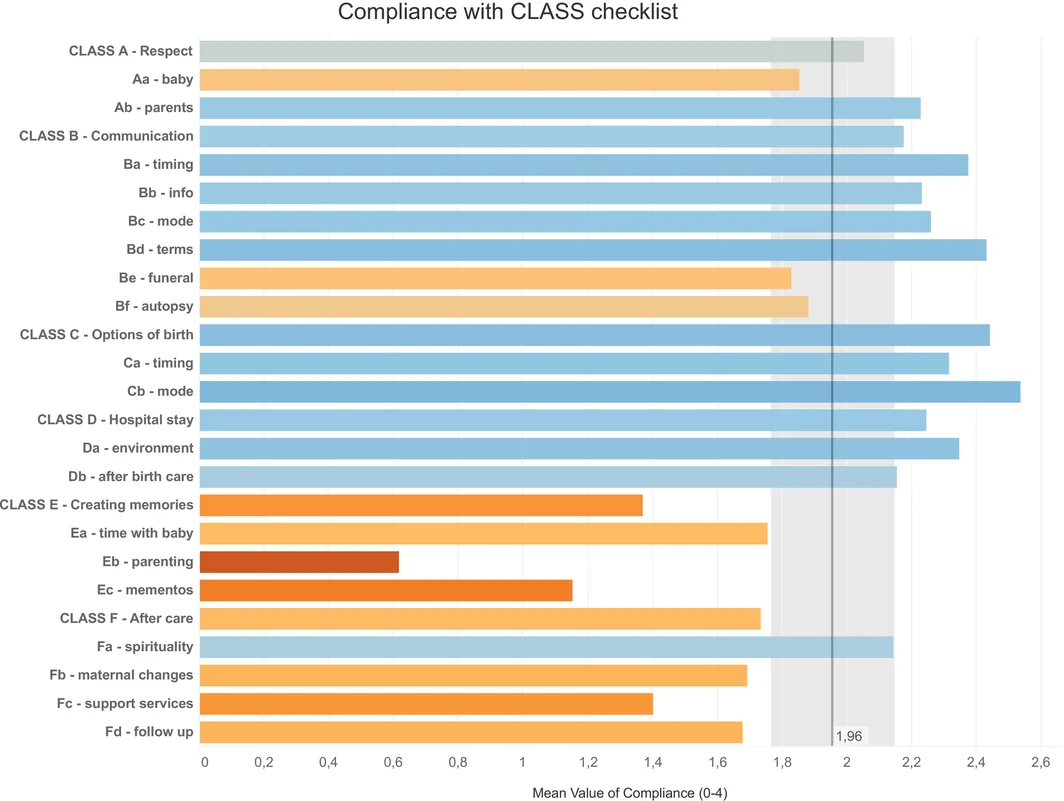
Bar chart showing adherence to the CLASS checklist. [Colour figure can be viewed at wileyonlinelibrary.com]

### 3.3 CLASS Checklist, Satisfaction, Respectful Care, and Psychological Outcomes

A linear relationship was observed between adherence to guideline items and psychological outcomes, indicating that higher adherence corresponded to improved parental well‐being. Specifically, a higher mean score on the CLASS checklist was associated with a lower score on PGS and NSESSS (Figure 3a). The same association was observed considering the number of items of the CLASS checklist: the more items were satisfied, the lower the intensity of grief (*p* < 0.0001) (Figure 3b) and post‐traumatic stress symptoms affecting mothers (*p* < 0.001). Figure 3 reports only the most representative data to avoid excessive complexity.

**FIGURE 3:**
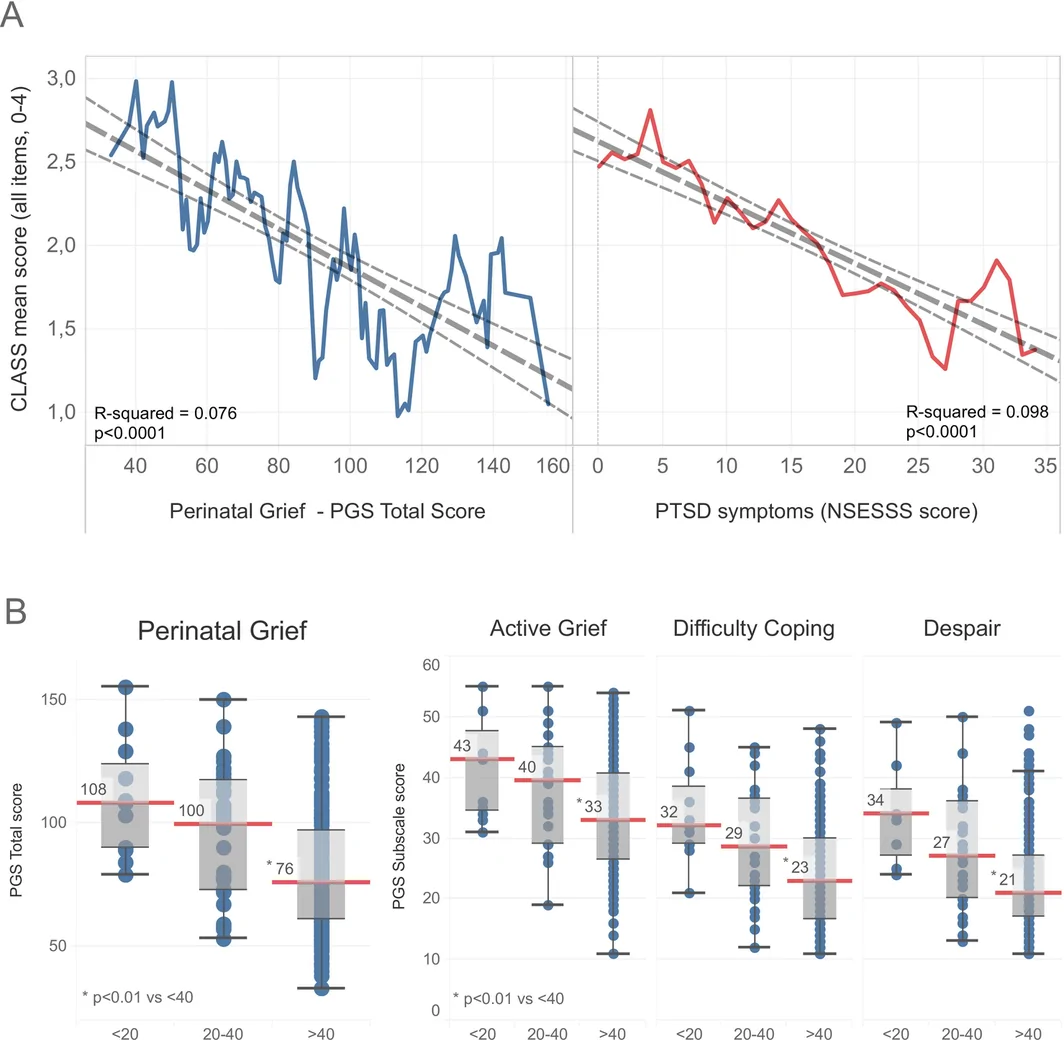
(A) Scatter plots showing the correlation between adherence to the CLASS checklist (expressed in terms of mean score) and the total score of PGS and NSESSS scales. (B) Box‐and‐whisker graphs showing the correlation between adherence to the CLASS checklist (expressed in terms of number of items satisfied) and the scores of PGS (total and subscales). [Colour figure can be viewed at wileyonlinelibrary.com]

In a univariate analysis, all items on the CLASS checklist were found to be associated with mothers' satisfaction with care (Figure S1). In a multivariate analysis (Figure 4), adherence to at least 40 CLASS checklist items was found to be an independent factor associated with greater satisfaction of care (OR 2.0, CI 1.1–3.8) and the perception of having received respectful care (OR 3.6, CI 1.9–7.0). A gestational age at loss between 20 and 25 weeks is positively associated with less satisfaction with care (OR 0.43, CI 0.2–0.9), whereas time after loss under 12 months is independently associated with higher satisfaction of care (OR 2.39, CI 1.1–4.9). Adherence to at least 40 CLASS checklist items was also found to be an independent factor related to a lower score on the PGS (OR 0.08, CI 0.1–0.2) and NSESSS (OR 0.21, CI 0.1–0.5). Also having a subsequent pregnancy after loss was related to a lower score on both scales (PGS—OR 0.20, CI 0.1–0.4; NSESSS—OR 0.30, CI 0.1–0.6). A sub‐analysis not shown here showed that location itself does not significantly impact outcomes.

**FIGURE 4:**
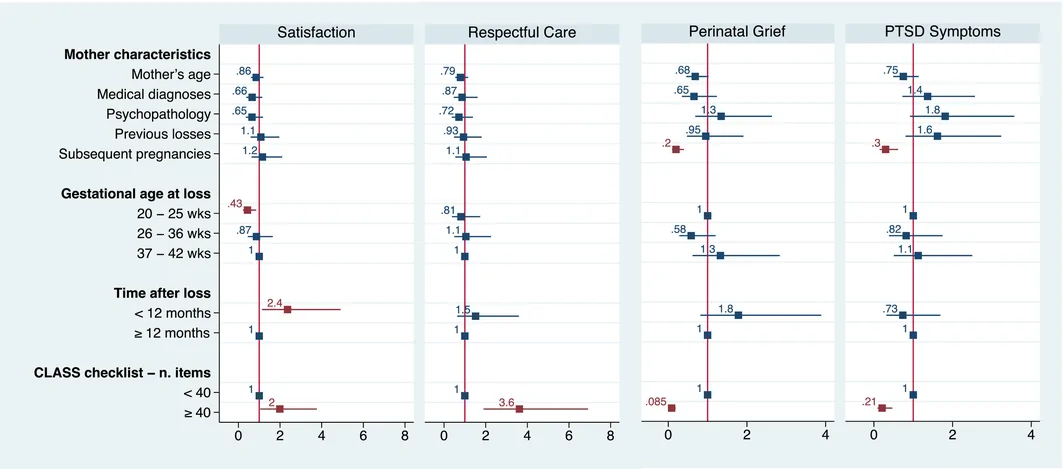
Forest plots showing the independent factors which influenced the satisfaction of care, the perception of respectful care, PGS score and NSESSS score. [Colour figure can be viewed at wileyonlinelibrary.com]

## 4 Discussion

### 4.1 A Snapshot of Bereavement Care

We found that the mean score of mothers' perception of adherence to guidelines was quite low with significant regional differences. Previous research, focused on HCPs' knowledge, identified a similar gap between Southern and Northern regions [19]. Such discrepancies confirm the urgency of improving formal training on bereavement care that is tailored to HCPs' needs. The most deficient categories were respect for the baby, communication about funeral arrangements and autopsy, creating memories, and aftercare. Creating memories and showing respect towards the baby, which are closely linked to mementoes and memories itself, are acknowledged to be healing practices that facilitate the expression of grief, and consequently, the grieving process [26]. However, this critical aspect was often neglected or discouraged by Italian HCPs. These findings corroborate data from a large international study conducted by Horey et al. (2021) which emphasized deficiencies in bereavement care especially around the practice of collecting memories [27].

Although aftercare services are crucial aspects of bereavement care pathways [28, 29], Italian HCPs lacked awareness about this issue. Insufficient specialized training and the absence of relevant Italian guidelines contribute to this situation [19, 30].

### 4.2 Adherence to Guidelines and Psychological Outcomes

The CLASS study effectively demonstrated that adherence to 40 or more CLASS checklist items was associated with increased satisfaction and perception of respectful care, as well as reduced levels of grief and posttraumatic stress symptoms. Similarly, a higher mean CLASS score correlates with better psychological outcomes for parents.

Our findings also highlighted two additional points: (1) lower satisfaction among women who experienced a perinatal loss before 25 weeks of gestational age, and (2) a decrease in satisfaction as time since the loss passes. We hypothesize that the first observation may be attributed to healthcare professionals potentially underestimating the psychological impact of an early loss on women [31]. Previous research has shown that insensitive comments addressed to grieving mothers by healthcare professionals reflect a lack of knowledge about the emotional aspects involved in early losses [32]. Regarding the second observation, the initial shock may begin to diminish in the days or months following the event. During this period, women may seek information about perinatal grief, thereby gaining a greater understanding of the support they lacked during their hospital stay. This increased awareness may, over time, contribute to a decrease in overall satisfaction.

### 4.3 Guidelines and Bereavement Care

The low level of adherence to international guidelines reported in our sample reflects the absence of official national guidelines and the variability of care across different regions and units. This finding is consistent with previous studies that have documented gaps and challenges in providing quality bereavement care in Italy [9].

Establishing national guidelines is a crucial first step to ensuring respectful care, as stated by the WHO [5, 16]. All HCPs involved in bereavement care should accurately implement these recommendations to ensure consistency of care [33]. The recent publication of Italian recommendations on the clinical management of stillbirth occurred after the data for this study were collected [17]. This timing allowed us to compare parents who received low‐quality care with those who received adequate care, all within similar social and cultural contexts, and even within the same hospital, as the quality of assistance often varied depending on the attending staff. In countries where national guidelines have been established for years, a more homogeneous quality of care may exist, making comparisons between different levels of assistance more challenging.

Contemporary bereavement care requires that HCPs possess fundamental knowledge in various disciplines, such as psychiatry, psychology, and sociology. However, this is not always the case. The CLASS checklist, which is easy to use and based on current literature, can significantly aid in providing respectful and supportive care. Furthermore, the CLASS study confirms the importance of international guidelines on bereavement care for both parents and healthcare professionals.

### 4.4 Implications

This study is the first to show, using clinical outcomes, a significant and proportional relationship between adherence to stillbirth care guidelines and the psychological well‐being of bereaved women, specifically regarding posttraumatic stress symptoms and grief levels. These results underscore the role of HCPs in safeguarding women's psychological well‐being. Preventing secondary traumatisation due to improper healthcare practices is vital, given the profound psychological effects of stillbirth on women, their families, and their marital relationships [34].

Therefore, we recommend the utilization of the CLASS checklist as a tool to gauge adherence to guidelines and enhance mental health following perinatal loss. This checklist serves as a resource for HCPs to evaluate and refine the quality of their care and identify areas needing additional training and support. Additionally, the CLASS checklist enables cross‐cultural comparisons and collaborations across various countries and settings by encapsulating the key components of major international bereavement care guidelines post‐stillbirth.

Finally, the CLASS study underscores the critical need for establishing official national guidelines in countries currently without them. Implementing such guidelines helps standardize care across facilities, ensuring consistent quality and equitable care for all bereaved women. Additionally, the development of national guidelines will elevate awareness and acknowledge stillbirth as a significant public health concern that warrants focused attention.

## 5 Strengths and Limitations

We initially considered the variety of bereavement care across Italy a potential limitation of this study; however, from a methodological perspective, it can be viewed as a strength. Inconsistencies in care allowed us to evaluate the impact of adherence to international guidelines on mothers' well‐being.

The wide distribution of the sample, in relation to the time elapsed since the loss, could contribute to recall bias among mothers who had a stillbirth many years ago. However, it is known that in cases of traumatic experiences, the coherence of memories is quite well preserved [35]. Furthermore, the wide distribution of this study allowed us to include more mothers who received different qualities of care. Over the years, improvements in Italian bereavement care have been documented [8, 9, 19], leading to observable differences in psychological outcomes among the sample. By conducting a multivariate analysis to adjust for the variable “time elapsed from loss,” we found no significant differences in psychological outcomes and care satisfaction among women more than 12 months post‐loss. This suggests that the effect of recall bias is minimal.

Another limitation is that our study focused on high‐income, westernized countries as the source of international guidelines, which may not capture the cultural diversity and specific needs of parents from different backgrounds. Nevertheless, the primary focus of the guidelines and the CLASS checklist is on the application of respectful care principles to stillbirth management. This approach ensures that every parent is treated with respect and that any cultural aspects are duly considered and respected.

Additionally, some limitations are inherent to the cross‐sectional study design. This methodology captures only a single point in time, making it difficult to establish causal relationships. While we can observe associations between guideline adherence and psychological outcomes, we cannot definitively determine if adherence to guidelines causes better psychological outcomes. Furthermore, we cannot assess changes over time or the long‐term impact of guideline adherence on psychological outcomes without longitudinal data.

In addition, our findings may not reflect the experiences of parents who did not have access to the internet or who were not reached by CiaoLapo organization via phone. Beyond the intentions of healthcare professionals, several issues could hinder the implementation of international guidelines in the Italian context. These aspects will be the focus of further research.

Finally, future research should include more diverse and representative samples of parents, as well as multiple methods, including longitudinal studies and qualitative analyses from various data sources, to provide a more comprehensive and accurate picture of bereavement care after stillbirth.

## 6 Conclusions

Our research provides critical insights into the experiences of women receiving bereavement care after stillbirth in Italy. We found low adherence to international guidelines for care following stillbirth. The CLASS checklist emerged as a valuable, user‐friendly, standardized tool that effectively evaluates and enhances care quality following a loss. Given the profound psychological impact of stillbirth on women and their families, it is crucial to ensure the consistent implementation of established care guidelines to prevent additional trauma. This study underscores the urgent need for the formulation and adoption of official national bereavement care guidelines in countries lacking them, including Italy, and emphasizes the importance of providing comprehensive training and support to healthcare professionals specializing in perinatal care.

## Funding

The did not receive any specific funding for this study.

## Ethics Statement

Ethics approval for the OPALE project was obtained by Florence University Ethics committee (n.175 prot 0261728 of 23/09/2021, n.189 prot 0333881 of 07/12/2021).

## Conflicts of Interest

The authors declare no conflicts of interest.

## Supporting information


**Figure S1:** Forest plots showing the association between CLASS checklist items and satisfaction of care.

**Table S1:** CLASS (CiaoLapo Stillbirth Support) Checklist items.

**Table S2:** Sociodemographic information of the sample.

## Data Availability

The data that support the findings of this study are available from the corresponding author upon reasonable request.
