# Supplementary material for: Adherence to Stillbirth Guidelines and Women's Psychological Well‐Being: The CLASS (CiaoLApo Stillbirth Support) Cross‐Sectional Study
Source: Birth. 2026 Jun 19;53(4):771–9. doi: 10.1111/birt.70087 (PMC13728504; doi:10.1111/birt.70087)
Supplement: Supplementary file 1 [file BIRT-53-771-s003.pdf]

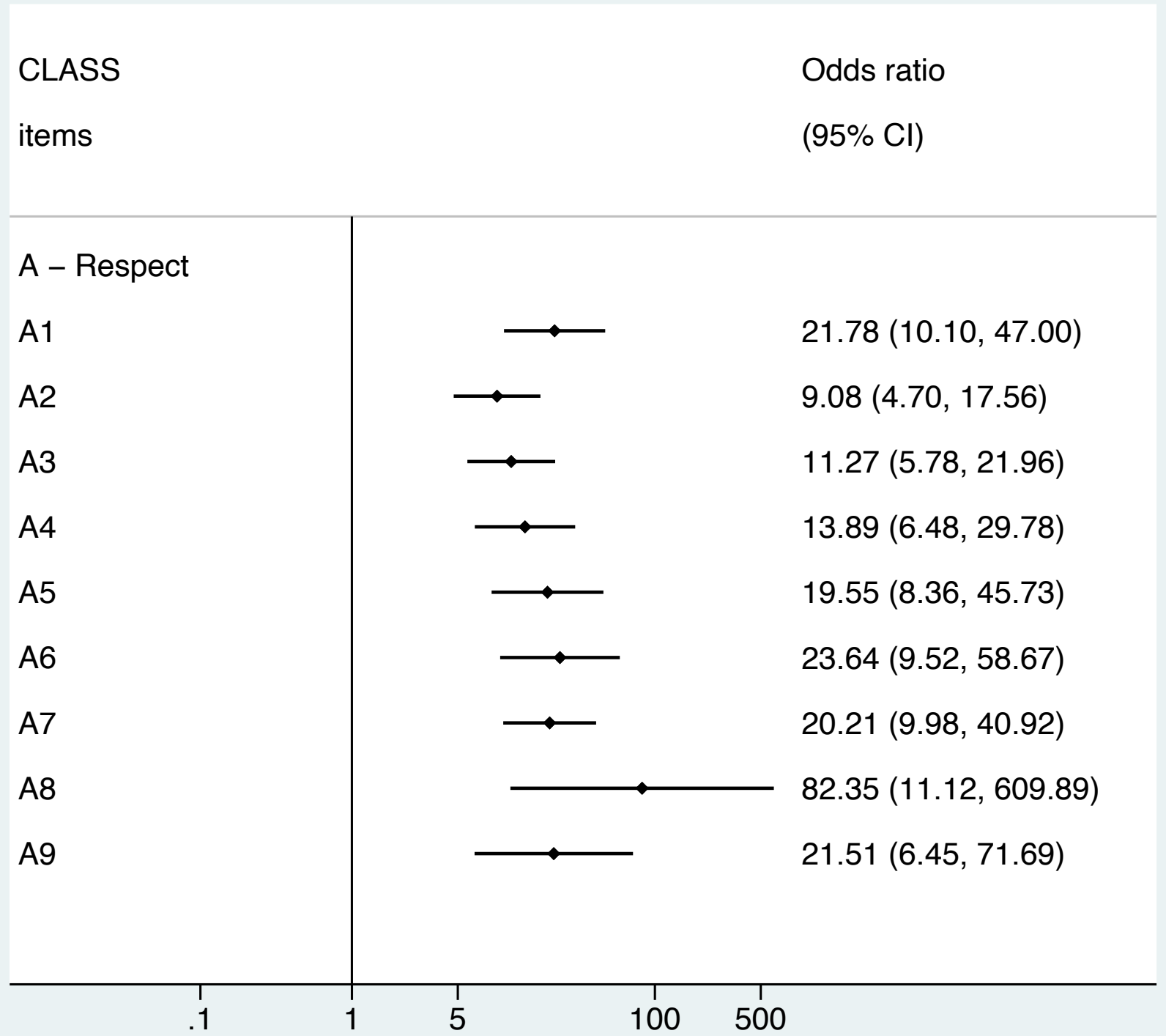
CLASS
items
Odds ratio
(95% CI)
A – Respect
A1 21.78 (10.10, 47.00)
A2 9.08 (4.70, 17.56)
A3 11.27 (5.78, 21.96)
A4 13.89 (6.48, 29.78)
A5 19.55 (8.36, 45.73)
A6 23.64 (9.52, 58.67)
A7 20.21 (9.98, 40.92)
A8 82.35 (11.12, 609.89)
A9 21.51 (6.45, 71.69)
.1 1 5 100 500

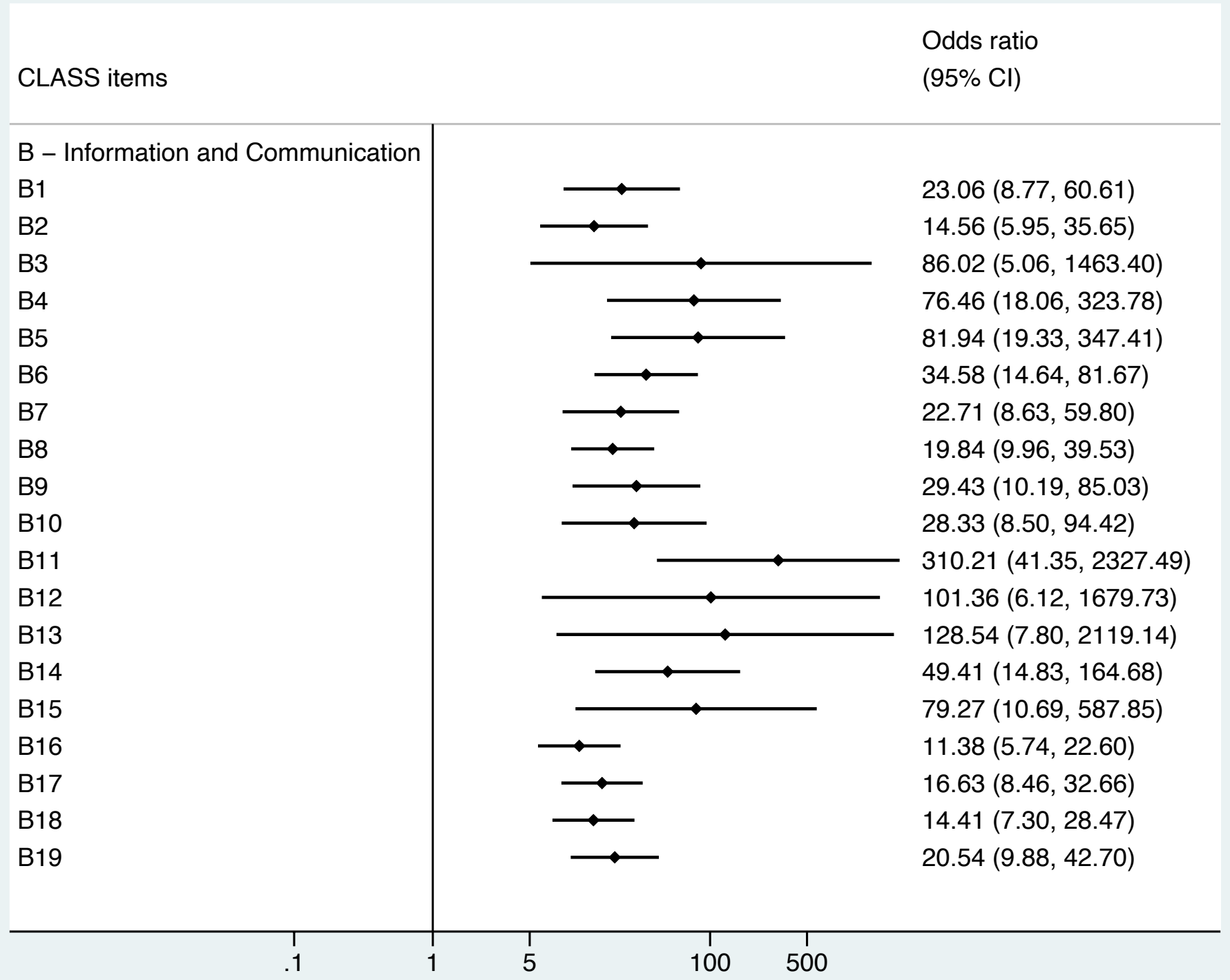
CLASS items
Odds ratio
(95% CI)
B – Information and Communication
B1 23.06 (8.77, 60.61)
B2 14.56 (5.95, 35.65)
B3 86.02 (5.06, 1463.40)
B4 76.46 (18.06, 323.78)
B5 81.94 (19.33, 347.41)
B6 34.58 (14.64, 81.67)
B7 22.71 (8.63, 59.80)
B8 19.84 (9.96, 39.53)
B9 29.43 (10.19, 85.03)
B10 28.33 (8.50, 94.42)
B11 310.21 (41.35, 2327.49)
B12 101.36 (6.12, 1679.73)
B13 128.54 (7.80, 2119.14)
B14 49.41 (14.83, 164.68)
B15 79.27 (10.69, 587.85)
B16 11.38 (5.74, 22.60)
B17 16.63 (8.46, 32.66)
B18 14.41 (7.30, 28.47)
B19 20.54 (9.88, 42.70)
.1 1 5 100 500

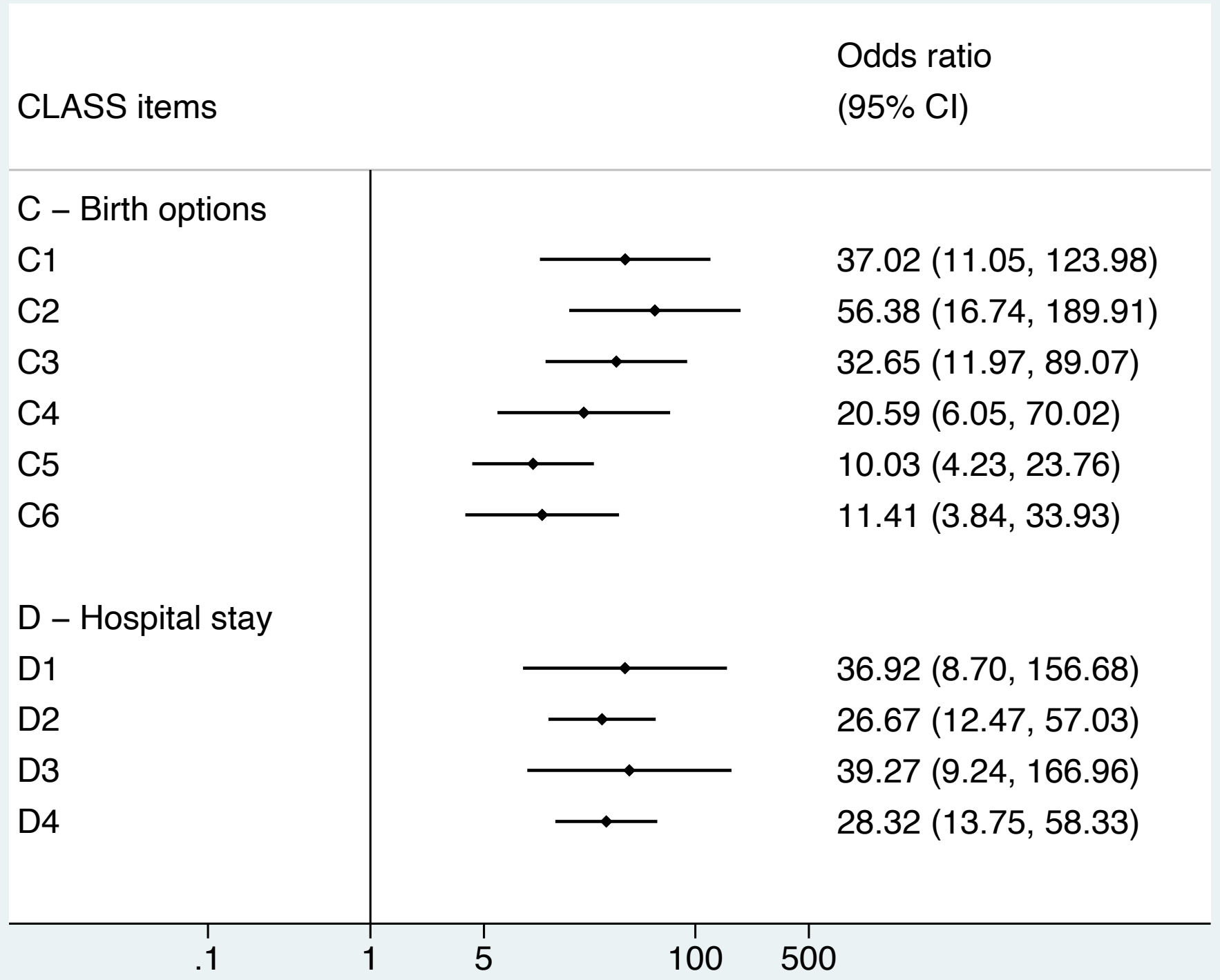
CLASS items
Odds ratio
(95% CI)
C – Birth options
C1 37.02 (11.05, 123.98)
C2 56.38 (16.74, 189.91)
C3 32.65 (11.97, 89.07)
C4 20.59 (6.05, 70.02)
C5 10.03 (4.23, 23.76)
C6 11.41 (3.84, 33.93)
D – Hospital stay
D1 36.92 (8.70, 156.68)
D2 26.67 (12.47, 57.03)
D3 39.27 (9.24, 166.96)
D4 28.32 (13.75, 58.33)
.1 1 5 100 500

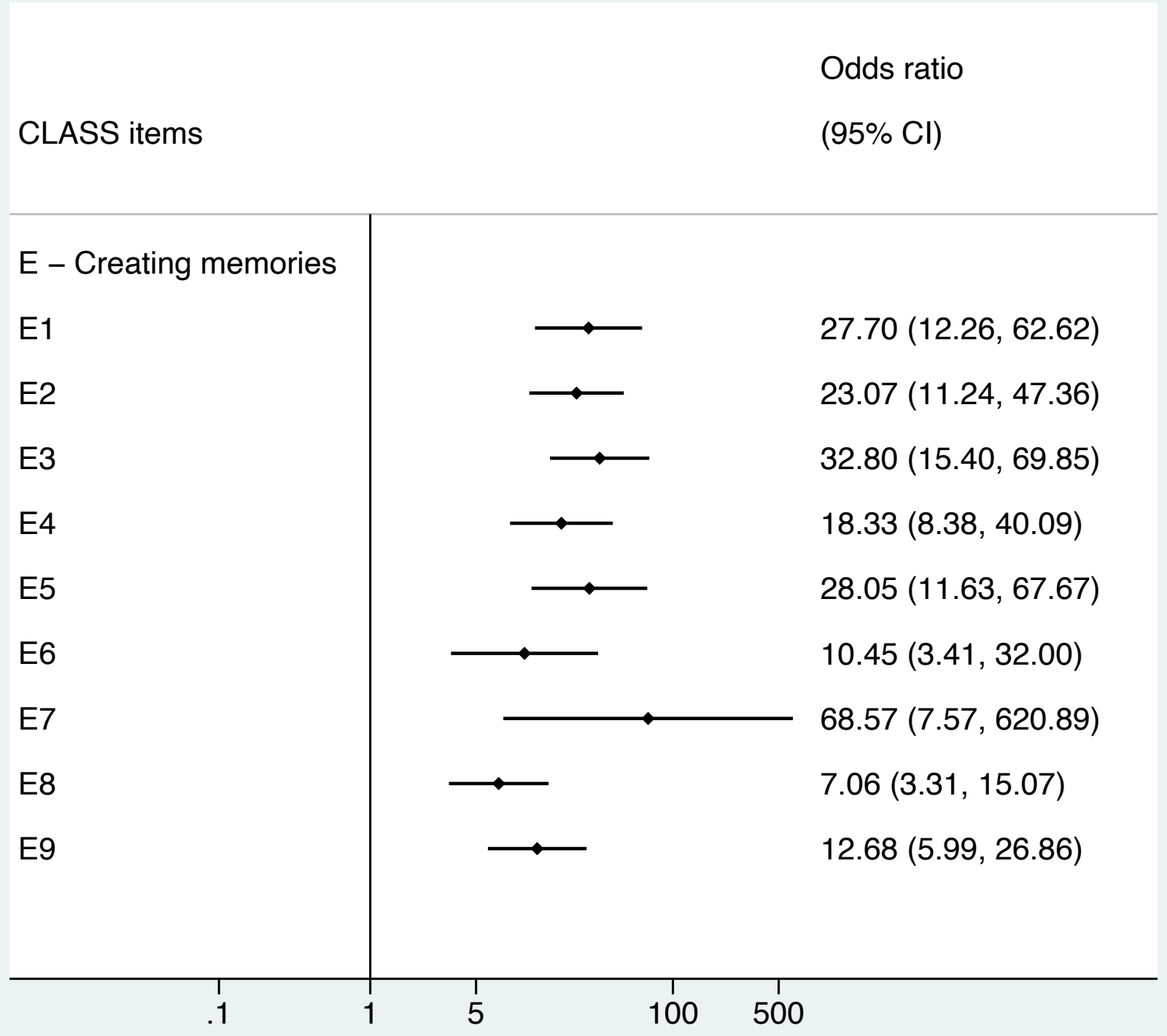
CLASS items
Odds ratio
(95% CI)
E – Creating memories
E1 27.70 (12.26, 62.62)
E2 23.07 (11.24, 47.36)
E3 32.80 (15.40, 69.85)
E4 18.33 (8.38, 40.09)
E5 28.05 (11.63, 67.67)
E6 10.45 (3.41, 32.00)
E7 68.57 (7.57, 620.89)
E8 7.06 (3.31, 15.07)
E9 12.68 (5.99, 26.86)
.1 1 5 100 500

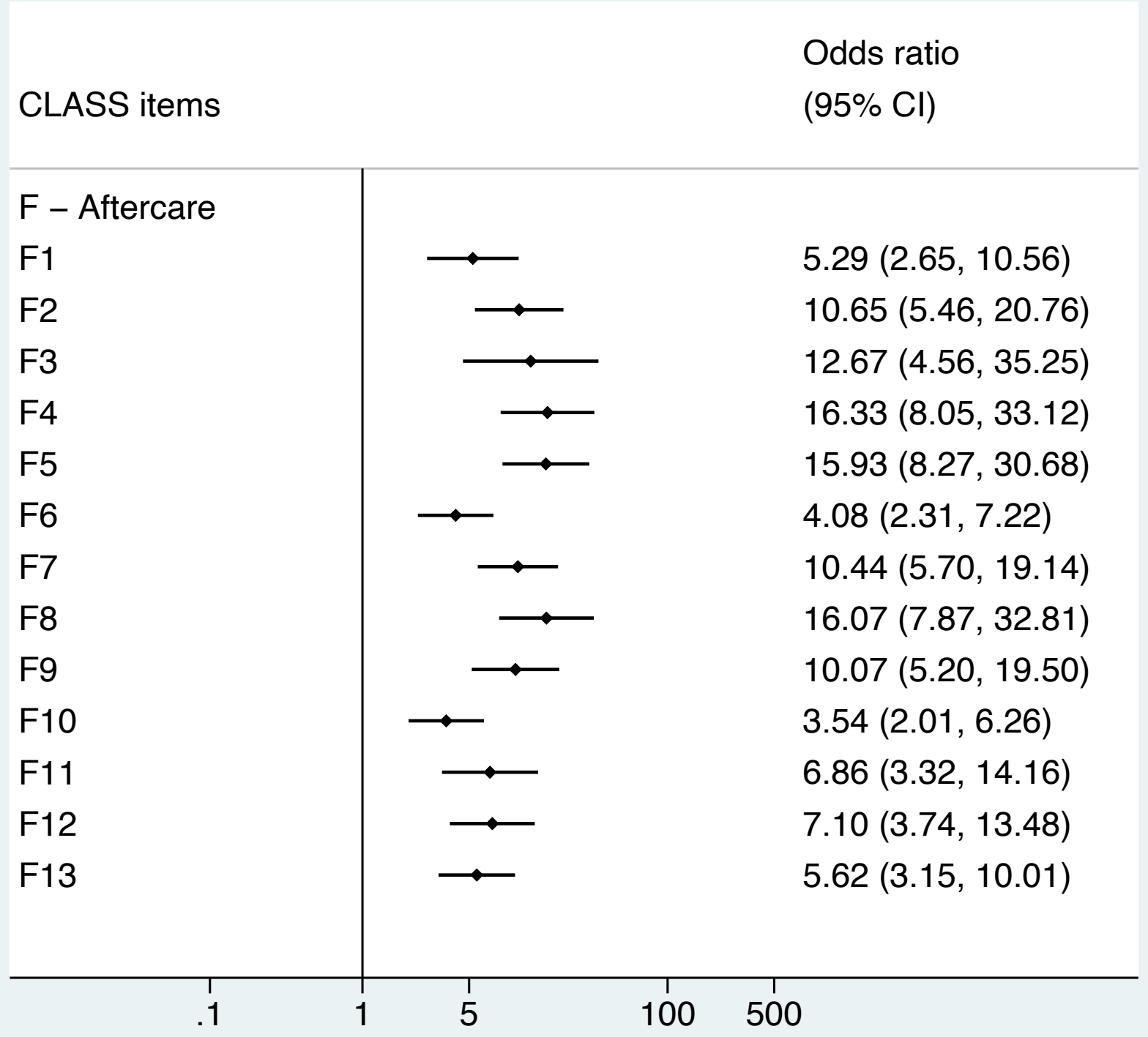
CLASS items
Odds ratio
(95% CI)
F – Aftercare
F1 5.29 (2.65, 10.56)
F2 10.65 (5.46, 20.76)
F3 12.67 (4.56, 35.25)
F4 16.33 (8.05, 33.12)
F5 15.93 (8.27, 30.68)
F6 4.08 (2.31, 7.22)
F7 10.44 (5.70, 19.14)
F8 16.07 (7.87, 32.81)
F9 10.07 (5.20, 19.50)
F10 3.54 (2.01, 6.26)
F11 6.86 (3.32, 14.16)
F12 7.10 (3.74, 13.48)
F13 5.62 (3.15, 10.01)
.1 1 5 100 500

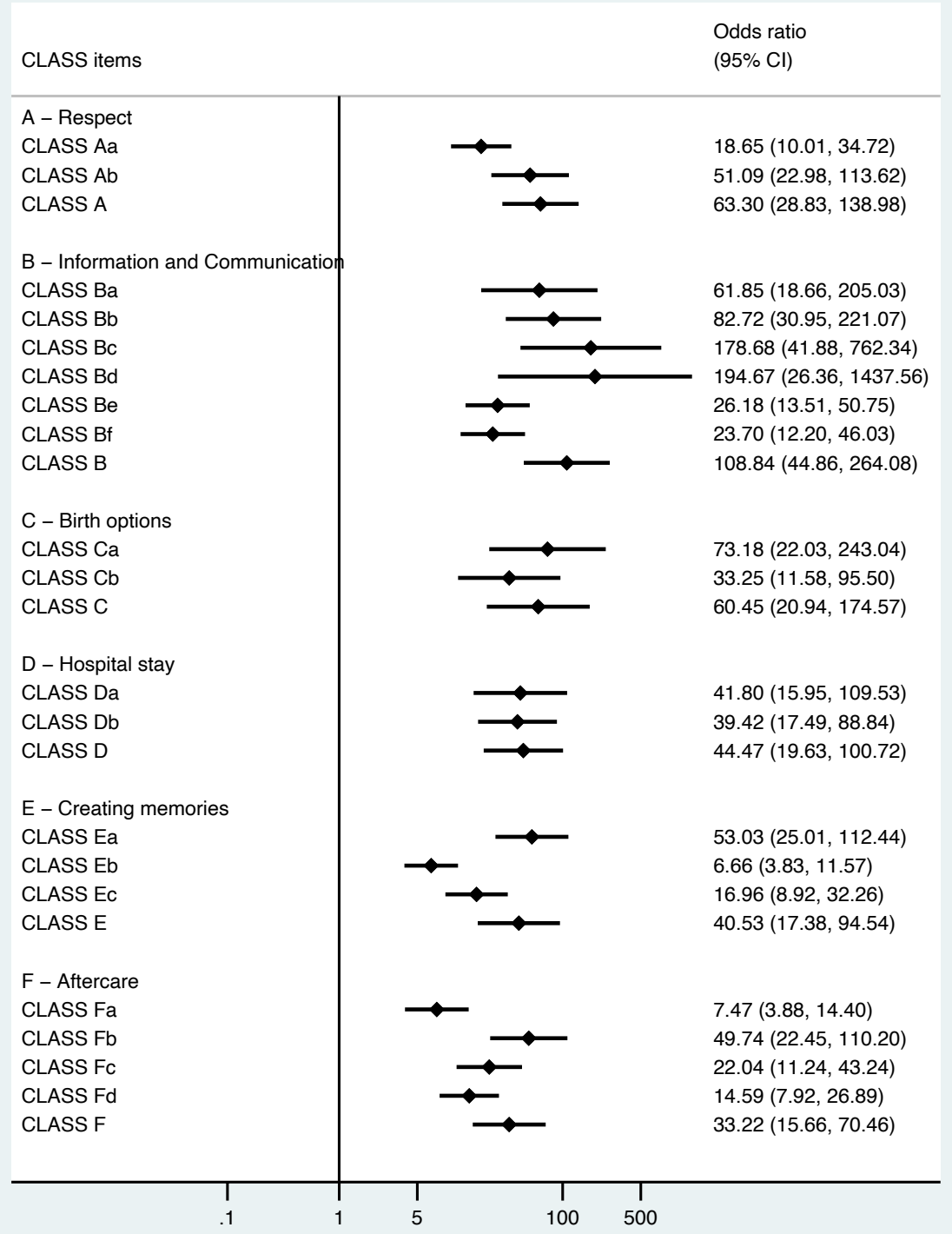
CLASS items
Odds ratio
(95% CI)
A – Respect
CLASS Aa 18.65 (10.01, 34.72)
CLASS Ab 51.09 (22.98, 113.62)
CLASS A 63.30 (28.83, 138.98)
B – Information and Communication
CLASS Ba 61.85 (18.66, 205.03)
CLASS Bb 82.72 (30.95, 221.07)
CLASS Bc 178.68 (41.88, 762.34)
CLASS Bd 194.67 (26.36, 1437.56)
CLASS Be 26.18 (13.51, 50.75)
CLASS Bf 23.70 (12.20, 46.03)
CLASS B 108.84 (44.86, 264.08)
C – Birth options
CLASS Ca 73.18 (22.03, 243.04)
CLASS Cb 33.25 (11.58, 95.50)
CLASS C 60.45 (20.94, 174.57)
D – Hospital stay
CLASS Da 41.80 (15.95, 109.53)
CLASS Db 39.42 (17.49, 88.84)
CLASS D 44.47 (19.63, 100.72)
E – Creating memories
CLASS Ea 53.03 (25.01, 112.44)
CLASS Eb 6.66 (3.83, 11.57)
CLASS Ec 16.96 (8.92, 32.26)
CLASS E 40.53 (17.38, 94.54)
F – Aftercare
CLASS Fa 7.47 (3.88, 14.40)
CLASS Fb 49.74 (22.45, 110.20)
CLASS Fc 22.04 (11.24, 43.24)
CLASS Fd 14.59 (7.92, 26.89)
CLASS F 33.22 (15.66, 70.46)
.1 1 5 100 500
