# Supplementary material for: Adherence to Stillbirth Guidelines and Women's Psychological Well‐Being: The CLASS (CiaoLApo Stillbirth Support) Cross‐Sectional Study
Source: Birth. 2026 Jun 19;53(4):771–9. doi: 10.1111/birt.70087 (PMC13728504; doi:10.1111/birt.70087)
Supplement: Supplementary file 2 [file BIRT-53-771-s001.docx]

**Supplementary Table 1.** CLASS (CiaoLapo Stillbirth Support) Checklist items.

# CLASS - CiaoLapo Stillbirth Support Checklist

| **A - Respect** | | |
| --- | --- | --- |
| **Aa - Baby** | | |
| **☐** | A1 | Name the baby |
| **☐** | A2 | Touch, bath and dress the baby |
| **☐** | A3 | Take pictures |
| **☐** | A4 | Put the baby in a cot in the same room of the parents |
| **Ab - Parents** | | |
| **☐** | A5 | Use a recognisable symbol for the staff, to denote bereavement |
| **☐** | A6 | Acknowledge cultural, religious and spiritual needs of parents |
| **☐** | A7 | Make repeated offers for holding the baby |
| **☐** | A8 | Provide privacy (but not abandoning) |
| **☐** | A9 | Allow women to spend time with their partner (or person of choice) before and after birth |
|  |  |  |
| **B -Information and Communication** | | |
| **Ba - Timing** | | |
| **☐** | B1 | Discuss issues at most appropriate time |
| **☐** | B2 | Choose a quiet and private environment |
| **☐** | B3 | If the mother is unaccompanied, offer to contact the partner or a friend |
| **Bb - Delivery of information** | | |
| **☐** | B4 | Be clear, honest, and sensitive |
| **☐** | B5 | Repeat important information |
| **☐** | B6 | Assure parents that it is normal to feel uncomfortable |
| **☐** | B7 | Do not avoid questions |
| **Bc - Mode of information** | | |
| **☐** | B8 | Give written information |
| **☐** | B9 | Be comfortable showing emotions |
| **☐** | B10 | Answer questions honestly |
| **☐** | B11 | Listen to parents |
| **☐** | B12 | Do not argue with parents |
| **Bd - Terminology** | | |
| **☐** | B13 | Use parent-friendly language (simple and straightforward) |
| **☐** | B14 | Use sensitive terms (avoid dehumanising terms as ‘fetus’, ‘product of conception’ etc) |
| **☐** | B15 | Do not use medical jargon |
| **Be - Burial and funeral services** | | |
| **☐** | B16 | Inform parents on laws and regulations |
| **☐** | B17 | Provide parents help with funeral arrangements |
| **Bf - Post-mortem examination** | | |
| **☐** | B18 | Give verbal and written information on autopsy/histology |
| **☐** | B19 | Allow time for discussion issues regarding autopsy/histology |
|  |  |  |
| **C - Birth options** | | |
| **Ca -Timing** | | |
| **☐** | C1 | Inform in advance parents of factors relating to birth (when, how, etc) |
| **☐** | C2 | Allow parents time to make appropriate decisions regarding birth |
| **Cb - Mode of birth** | | |
| **☐** | C3 | Offer parents choices in birthing options (when available) |
| **☐** | C4 | Offer parents the option of returning home (if possible) |
| **☐** | C5 | Always offer obstetric analgesia |
| **☐** | C6 | Avoid using sedation |
|  |  |  |
| **D - Hospital stay** | | |
| **Da - Environment** | | |
| **☐** | D1 | Offer parents the choice of the less distressing ward (possibly provide a private room) |
| **☐** | D2 | Always allow to spend time with the baby |
| **Db - After-birth care** | | |
| **☐** | D3 | Do not urge parents to leave the hospital |
| **☐** | D4 | Offer continuity of care |
|  |  |  |
| **E - Creating memories** | | |
| **Ea - Spending time with baby** | | |
| **☐** | E1 | Clearly explain parents the length of time they are allowed to spend with their baby |
| **☐** | E2 | Let the parents all the time they need to stay with the baby |
| **☐** | E3 | Always offer parents to see and hold the baby, but do not force them; discuss what is the best option for them |
| **☐** | E4 | Respect their wishes regarding seeing and holding the baby |
| **☐** | E5 | Prepare parents for the appearance of the baby |
| **Eb - Parenting** | | |
| **☐** | E6 | Allow parents to bath and dress their baby |
| **☐** | E7 | Allow siblings to spend time with the baby |
| **Ec - Mementos** | | |
| **☐** | E8 | Take pictures of the baby and keep them in the hospital |
| **☐** | E9 | Provide parents with mementos (lock of hair, hand and footprints, ID bracelet, cot card, pictures) |
|  |  |  |
| **F - Aftercare** | | |
| **Fa - Spiritual / religious needs** | | |
| **☐** | F1 | Allow parents to bless, baptize or perform any other religious ceremony for their baby |
| **Fb - Maternal changes** | | |
| **☐** | F2 | Inform mothers on milk production |
| **☐** | F3 | Offer a consultation with a lactation consultant |
| **☐** | F4 | Inform mothers on bleeding |
| **☐** | F5 | Inform mothers on psychological consequences of perinatal loss |
| **☐** | F6 | Provide a follow-up at 6 weeks post birth. Give name and number of the case manager |
| **Fc - Support services** | | |
| **☐** | F7 | Provide early psychological support |
| **☐** | F8 | Provide written information on support services (include fathers) |
| **Fd -Follow-up** | | |
| **☐** | F9 | Provide name and number of the case manager |
| **☐** | F10 | Provide an appointment with a senior obstetrician within 2 months |
| **☐** | F11 | Where possible, use rooms away from the hospital |
| **☐** | F12 | Give and explain results of post-mortem examinations |
| **☐** | F13 | Discuss implication for future pregnancies |
